# Molecular Mechanisms of Foreign Body Responses to Neural Electrodes and Surface Biofunctionalization Strategies for Interface Modulation

**DOI:** 10.3390/ijms27156752

**Published:** 2026-07-28

**Authors:** Ziliang He, Junlong Ma, Yun Liu, Zhanhong Du

**Affiliations:** 1 Department of Biomedical Engineering, Southern University of Science and Technology, Shenzhen 518055, China; 12433439@mail.sustech.edu.cn; 2 Faculty of Life and Health Sciences, Shenzhen University of Advanced Technology (SUAT), Shenzhen 518107, China; 3 Brain Cognition and Brain Disease Institute, Shenzhen Institute of Advanced Technology (SIAT), Chinese Academy of Sciences (CAS), Shenzhen 518055, China; majlong@mail3.sysu.edu.cn; 4 The First Affiliated Hospital of Nanjing Medical University, Nanjing 210029, China; liuyun@njmu.edu.cn; 5 Jiangsu Brain-Computer Interface Research Institute, Nanjing 210033, China; 6 Shenzhen-Hong Kong Institute of Brain Science-Shenzhen Fundamental Research Institutions, Shenzhen 518055, China

**Keywords:** neural electrodes, neural interfaces, foreign body response, neuroinflammation, microglia, astrocytes, surface biofunctionalization, drug delivery, biofunctional materials

## Abstract

Long-term implantable neural electrodes underpin brain–machine interfaces, deep brain stimulation, epilepsy monitoring, and closed-loop neuromodulation. Following chronic implantation, however, the foreign body response (FBR) at the electrode–tissue interface remains a major constraint on long-term performance, as reflected by increased interfacial impedance, lower signal-to-noise ratios, fewer resolvable units, and higher stimulation thresholds. This deterioration arises from interrelated events that include implantation injury, protein adsorption, blood–brain barrier disruption, complement activation, glial reactivity, oxidative stress, glial scar formation, and neuronal loss. It cannot be attributed solely to material ageing or encapsulation failure. This review examines the molecular mechanisms of neural-electrode FBR and relates them to surface-biofunctionalization strategies, including antifouling coatings, bioactive ligands, immobilized neurotrophic factors, drug-eluting electrodes, and emerging immunomodulatory interfaces. Establishing mechanistic links among molecular events, material interfaces, and functionalization strategies may guide the rational design of durable neural electrodes.

## 1. Introduction

### 1.1. Clinical and Technical Significance of Long-Term Stability of Neural Electrodes

Implantable neural electrodes mediate information exchange between electronic systems and neural tissue [1,2,3]. In clinical practice, deep brain stimulation (DBS) is used to treat Parkinson’s disease, essential tremor, and dystonia, whereas electrocorticography (ECoG) and depth electrodes support epileptic-focus localization and preoperative assessment. In research and translational settings, high-density microelectrode arrays enable neural recording, movement-intention decoding, neuroprosthetic control, and closed-loop neuromodulation [4,5]. Across these applications, durable electrical performance and an acceptable tissue response are essential in the complex cerebral microenvironment.

Long-term stability remains a central bottleneck in translating neural interfaces from acute experiments to chronic use. Chronic neural electrodes must maintain low impedance, a high signal-to-noise ratio (SNR), and reliable stimulation efficiency for months or years. Although many devices perform well soon after implantation, recording quality, stimulation efficiency, and the number of functional channels often decline over time [6]. This decline shortens device lifetime and may increase the need for reoperation and long-term management. Traditionally, electrode failure has been attributed to material ageing, packaging defects, or electrochemical degradation. Increasing evidence indicates, however, that sustained molecular, cellular, and mechanical interactions between the implant and surrounding tissue also shape the long-term electrode–tissue interface [2,7,8]. Long-term stability should therefore be considered at both the device-engineering and tissue-interface levels.

From a clinical and translational perspective, long-term electrode stability is not simply a matter of extending the lifetime of an individual device. It determines whether experimental platforms can become reliable, maintainable chronic technologies. For closed-loop neuromodulation and brain–machine interfaces, recording stability governs whether decoding algorithms can be transferred across days, weeks, and years. For stimulation systems, changes in interfacial impedance and threshold directly affect energy consumption, selectivity, and safety. If interface responses progressively eliminate functional channels, even highly adaptive downstream algorithms cannot fully compensate for degradation of the signal source [9,10]. Electrode failure is therefore a systems-level challenge spanning materials science, tissue engineering, and algorithm design.

### 1.2. Chronic Signal Attenuation and Foreign Body Response

Chronic deterioration in neural-electrode performance is commonly first detected as persistent changes in electrical parameters and signal quality. Impedance near 1 kHz at the recording site can increase over time, increasing thermal noise and reducing the signal-to-noise ratio [2,11]. Neuronal spike amplitudes may decline while background noise increases, progressively compromising the separability of neural signals [12]. Neuronal loss, glial encapsulation, and greater electrode-to-neuron distance further reduce the number of resolvable units per channel and can ultimately cause channel failure [13,14]. For stimulation electrodes, higher current or voltage thresholds to achieve a comparable neuromodulatory effect increase energy demand, broaden stimulation fields, and may introduce adverse effects [15,16].

During long-term intracranial implantation, electrode performance is affected not only by material ageing or encapsulation failure but also by neuroinflammation and scar formation. The principal biological process is implant-induced foreign body response (FBR), a host response to non-self materials that has a distinct cellular composition and time course in the central nervous system. Mechanical injury and microvascular rupture during implantation disrupt the blood–brain barrier (BBB) [17]. Blood- and cerebrospinal-fluid-derived proteins subsequently adsorb to the electrode surface, forming a conditioning layer whose composition and conformation influence subsequent immune recognition. Microglia then migrate towards and become activated at the interface, while peripheral monocytes and macrophages may enter the parenchyma through the compromised BBB. Persistent inflammatory signalling promotes reactive astrocytosis and extracellular-matrix deposition, ultimately forming a glial scar around the electrode [17,18,19].

FBR comprises temporally distinct but mutually reinforcing phases, progressing from early injury to subacute inflammatory amplification and chronic tissue remodelling [20,21]. In the acute phase, bleeding, protein adsorption, and complement activation shape initial immune recognition. During the subacute phase, interactions among microglia, macrophages, and astrocytes influence whether inflammation resolves. In chronic stages, extracellular-matrix (ECM) deposition, neuronal loss, and electrochemical changes at the interface contribute to persistent functional decline. These processes interact across time; separating them conceptually can obscure the effects of early molecular events on long-term signal quality [22,23].

### 1.3. Molecular Target-Functionalization Strategy-Interface Outcome Analysis Framework

Previous reviews of neural-electrode stability have generally been organized by material class or device architecture, including conductive polymers, carbon-based materials, hydrogels, flexible electrodes, and ultramicroelectrodes [2,14]. Although useful for material selection and device engineering, this organization provides limited systematic analysis of the molecular targets engaged by specific material strategies and their mechanisms of action. Advances in neuroimmunology, biomaterials, and spatial omics have shifted the study of electrode–tissue interfaces beyond conventional histology towards cellular subpopulations, signalling pathways, and gene-expression networks. Transcriptomic studies indicate that chronic implant interfaces involve extensive differential gene expression associated with inflammatory amplification, complement activation, extracellular-matrix remodelling, and neuronal injury [21,24,25]. A molecularly centred analytical framework is therefore needed alongside material- and device-based perspectives.

**Figure 1:**
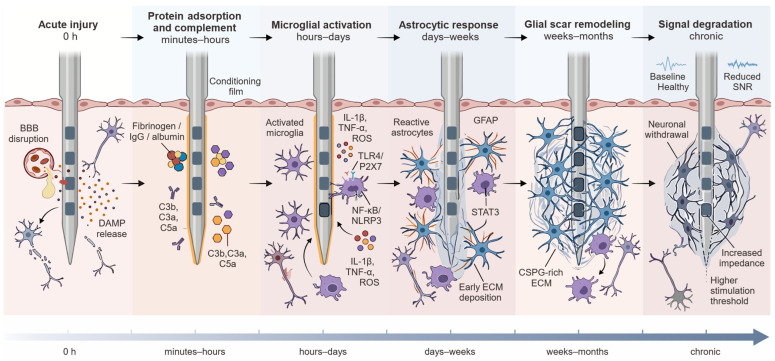
Temporal cascade of foreign body responses at the neural electrode–tissue interface. The scheme summarizes the progression from implantation injury, protein adsorption and complement activation to glial activation, scar remodeling and chronic signal degradation. The lower panel highlights molecular drivers and functional readouts relevant to long-term neural electrode performance. Color and arrow key: colors distinguish temporal phases; arrows indicate chronological progression and mechanistic links.

This review applies a molecular-target–surface-biofunctionalization–interface-outcome framework to relate key molecular nodes to functionalization strategies. TLRs, NLRP3, STAT3, and CSPGs participate in early immune recognition, inflammasome activation, astrocyte reactivity, and inhibitory matrix deposition, respectively. Antifouling coatings, bioactive ligands, neurotrophic-factor immobilization, drug delivery, and immunomodulatory interfaces can influence protein adsorption, complement deposition, glial reactivity, neuronal survival, and inflammatory resolution. The temporal cascade of FBR provides the basis for matching each intervention to its most relevant stage of implantation. Figure 1 depicts the continuum from acute injury to chronic signal attenuation.

Surface biofunctionalization should not be understood in a narrow sense as a passive coating modification attached to the outer layer of the electrode, but should be regarded as a molecular engineering strategy that can actively directionally regulate the interface microenvironment. Different functionalized interfaces can respectively play a role in initial protein adsorption and complement recognition, local signal exchange between microglia and astrocytes, neuronal survival and close coupling, as well as interface stability under long-term mechanical micromotion and electrochemical working conditions. Not every strategy above must be fully satisfied at the same time, but the degree of synergy determines whether short-term histological improvement can be further translated into long-term electrophysiological stability [12,26].

## 2. Molecular Mechanisms of Foreign Body Responses to Neural Electrodes

The interface response after neural electrode implantation has a well-defined temporal evolution and involves multiple components such as blood vessels, immune cells, glial cells, neurons and extracellular matrix [17,18]. The discussion at the mechanistic level can start from the early events of implantation and sequentially connect protein adsorption, complement activation, microglia and astrocyte responses, oxidative stress, neuronal damage and pro-repair immune regulation, thereby laying the foundation for target selection for surface biofunctionalization strategies [20,27].

This mechanistic framework also explains why material strategies that perform well in vitro often show limited efficacy after chronic implantation in vivo. In vitro assays generally capture local endpoints, such as cell adhesion, viability, or short-term cytokine release. By contrast, the implanted brain also undergoes vascular disruption, exchange of cerebrospinal-fluid and plasma proteins, immune-cell migration, glial-network remodelling, electrode micromotion, and stimulation-related electrochemical perturbation. Neural-electrode interfaces should therefore be understood as multicellular, time-dependent, and multiphysical systems [19,28].

### 2.1. Implantation Injury, Protein Adsorption and Blood–Brain Barrier Disruption

Neural-electrode implantation inevitably causes local tissue injury. Probe insertion can sever neuronal and glial processes, disrupt microvessels, and produce focal bleeding, ischaemia, and mechanical stress [26]. Vascular injury compromises BBB integrity, permitting plasma proteins and immune mediators that are normally confined to the circulation to enter the parenchyma. The BBB is maintained by endothelial cells, pericytes, the basement membrane, and astrocytic endfeet. Its disruption alters local exchange and establishes the conditions for subsequent immune recognition and inflammatory amplification [17,18,27].

Plasma proteins and cerebrospinal fluid proteins entering the interface microenvironment will be adsorbed on the electrode surface in a short period of time, forming a so-called conditioning film [19,20]. The host cells do not directly recognize the “bare” material surface, but interact with this layer of adsorbed proteins. The type, quantity, conformation and spatial distribution of protein adsorption are affected by factors such as material hydrophilicity and hydrophobicity, surface energy, charge, roughness and chemical composition. Common adsorbed proteins include albumin, fibrinogen, immunoglobulins, and various complement proteins [2,28,29].

Fibrinogen is particularly important in early interface responses. Fibrinogen adsorbed on specific surfaces may undergo conformational changes, exposing epitopes that can be recognized by integrins such as Mac-1/CD11b/CD18, thereby promoting the adhesion and activation of microglia and macrophages. At the same time, damage-associated molecular patterns (DAMPs) such as ATP and HMGB1 released by damaged cells can form danger signals together with adsorbed proteins. Therefore, the surface properties of the material indirectly determine the intensity and direction of the subsequent innate immune response by affecting the molecular composition, conformational state and spatial distribution of the protein layer [17,19].

The protein-modulating membrane also allows the “chemical identity” on the material surface to be quickly converted into a “biological identity” after implantation. The interfaces that cells actually come into contact with are often already covered by albumin, fibrinogen, immunoglobulins, complement components, and extracellular matrix fragments, whereby the effects of the original metal, polymer, or nanomaterial surface are re-presented via the adsorbed protein layer. The composition of this molecular layer rearranges over time, local inflammatory status, and changes in material surface properties, and continues to affect integrin recognition, complement deposition, and immune cell adhesion. Although early protein adsorption occurs within the first few minutes to hours after implantation, it often affects the molecular direction of subsequent FBR [18,19,20].

### 2.2. Complement Activation and Early Innate Immune Response

The complement system links early protein adsorption to innate immune activation. It comprises plasma and membrane-bound proteins that are activated through the classical, lectin, and alternative pathways. The classical pathway is usually initiated by antibodies or C1q-containing recognition complexes, the lectin pathway by carbohydrate-recognition structures such as mannose-binding lectins, and the alternative pathway by spontaneous C3 hydrolysis and amplification on foreign surfaces. At implant interfaces, the alternative pathway is particularly relevant because it can be activated directly on materials that lack host complement regulators [19,28].

C3 cleavage is central to complement activation [30,31]. In the alternative pathway, hydrolysed C3 [C3(H_2_O)] binds factor B, which is then cleaved by factor D to generate C3 convertase. This enzyme amplifies the production of C3a and C3b. Surface-deposited C3b acts as an opsonin that promotes immune recognition, whereas C3a and C5a function as anaphylatoxins and chemoattractants. Through receptors including C3aR and C5aR1 on microglia, astrocytes, endothelial cells, and peripheral immune cells, these mediators can enhance inflammatory-cell recruitment and cytokine release and may increase vascular permeability, further aggravating BBB disruption [2,17].

The importance of the complement pathway in neural electrode interfaces is also reflected in its amplification characteristics. A small amount of C3b deposition can form a positive feedback through alternative pathways, transforming protein adsorption events originally limited to the material surface into an inflammatory network with chemotactic and cell activation effects. C3a and C5a not only recruit and activate microglia and peripheral immune cells, but may also affect endothelial permeability and astrocyte responses. Complement thus becomes an important molecular coupling between protein adsorption, vascular responses, and glial inflammation.

### 2.3. Microglial Activation and Pro-Inflammatory Signaling Pathways

Microglia are resident immune cells in the central nervous system (CNS) and the main effector cells in neural electrode FBR [32,33]. Under steady-state conditions, microglia monitor the surrounding microenvironment through highly dynamic protrusions. Implantation damage, DAMPs, adsorbed protein layers, complement fragments and changes in the local ionic environment can all cause it to shift from a surveillance state to an activated state. After activation, the morphology of microglia gradually changes from branched to hypertrophic or amoeba-like, and chemotactically aggregates towards the electrode interface [27,34].

Early studies commonly described microglial activation using an M1/M2 framework; this dichotomy is now considered inadequate for the complex states observed after chronic implantation. Microglia instead occupy a continuum of pro-inflammatory, phagocytic, anti-inflammatory, and repair-associated states. In neural-electrode FBR, acute injury and chronic mechanical stimulation can sustain pro-inflammatory programmes, cytokine release, and glial reactivity [33,35]. Because implanted electrodes are non-degradable and exceed the size that a single phagocyte can engulf, microglia and infiltrating macrophages may enter persistent frustrated phagocytosis. This incomplete clearance can sustain cytoskeletal rearrangement, lysosomal stress, reactive oxygen species (ROS) production, and NF-κB-associated transcriptional activity, thereby linking acute injury to chronic interface inflammation [34].

The pro-inflammatory response of microglia consists of multiple mutually coupled signaling modules, among which TLR/NF-κB, MAPK, NLRP3 inflammasome and ROS-related pathways are the most representative. In the pro-inflammatory signaling network, the Toll-like receptor (TLR) pathway is an early activated pattern recognition system [33,35]. TLRs are pattern recognition receptors (PRRs) that can recognize exogenous pathogen-associated molecular patterns (PAMPs) and endogenous DAMPs. At the neural electrode interface, TLR2 and TLR4 are closely related to adsorbed proteins, cell debris and sterile damage signals. After receptor activation, downstream signaling complexes are usually recruited through myeloid differentiation factor 88 (MyD88), thereby activating the NF-κB (nuclear factor-kappa B) pathway [36]. After entering the nucleus, NF-κB induces the expression of TNF-α, IL-1β, IL-6 and various chemokines, and is the core transcriptional regulatory node in the pro-inflammatory cascade [37,38].

The NLRP3 inflammasome is a key platform connecting intracellular danger signals and the mature release of IL-1β [34,35]. At the electrode interface, ATP released from damaged cells can activate P2X7 receptors, trigger potassium ion efflux and promote NLRP3 assembly. NLRP3 then recruits ASC and Caspase-1, and activated Caspase-1 cleaves pro-IL-1β and pro-IL-18 into their mature forms. Mature IL-1β can further amplify local inflammatory responses and participate in astrocyte responses and neuronal damage [36,38].

Oxidative stress permeates the inflammatory network. Activated microglia generate ROS through NADPH oxidase, mitochondrial dysfunction, and related pathways. ROS directly damage lipids, proteins, and DNA, while also activating NF-κB, MAPK, and the NLRP3 inflammasome. This reciprocal amplification between inflammation and oxidative stress contributes to persistent interface responses [35,36].

The sustained activation of pro-inflammatory pathways creates a pro-inflammatory microenvironment around the electrode and drives glial scarring and neuronal damage. Local drug delivery targeting nodes such as NF-κB, NLRP3 or ROS thus constitutes an important intervention direction for functionalized electrodes [2,14]. At the same time, flexible materials and minimally invasive implant techniques can reduce upstream activation by reducing mechanical stimulation. In addition to microglia, peripheral monocytes/macrophages and perivascular macrophages (PVMs) that enter the brain parenchyma through the damaged BBB may also participate in local inflammatory responses and blood vessel-related interface remodeling [20,39].

During chronic implantation, the spatial distribution of microglial responses is also informative. Cells adjacent to the electrode experience more direct material contact, protein adsorption, and micromotion, whereas cells at greater distances are more strongly influenced by diffusible cytokines, chemokines, and oxidative stress. Averaging a single tissue section can therefore obscure the most active regions of the interface response. Whenever possible, evaluations of functionalization strategies should incorporate distance-stratified analyses, spatial transcriptomics, or multiplexed immunoimaging to distinguish near-field material effects from secondary tissue responses.

### 2.4. Astrocyte Reaction and Glial Scar

Astrocytes are abundant and functionally diverse glial cells in the CNS that can undergo reactive astrogliosis under injury and inflammation. After neural electrode implantation, signals such as TNF-α and IL-1β released by microglia can induce morphological hypertrophy, migration, and proliferation of astrocytes, and upregulate intermediate filament proteins such as glial fibrillary acidic protein (GFAP), vimentin, and nestin [40].

Reactive astrocytes gather toward the electrode interface and form glial scars together with microglia, infiltrating immune cells, and extracellular matrix. This process includes multiple levels such as structural packaging, inflammatory factor response, mechanical signal transduction and matrix remodeling [40,41].

In the astrocyte response, the JAK/STAT3 pathway is an important regulatory axis connecting the stimulation of inflammatory factors and the expression of scar-related genes. JAK is a type of tyrosine kinase coupled to cytokine receptors, and STAT3 is its downstream transcription factor. Signals such as IL-6 family cytokines can activate JAK and promote STAT3 phosphorylation; phosphorylated STAT3 forms dimers and enters the nucleus, driving the expression of GFAP and a variety of genes related to astrogliosis, migration and scar formation [40,42].

The complexity of glial scars is that they are both protective and inhibitory. A modest astrocyte response helps limit damage spread, restore ion homeostasis, and isolate necrotic tissue; but when the response persists, the GFAP-positive cell layer and CSPG-enriched ECM form a structural and chemical barrier that makes it difficult for neurites to re-approach the recording site. For neural electrodes, a more appropriate design goal is to avoid excessive scar densification and long-term inhibitory ECM accumulation while retaining necessary tissue repair and barrier functions [43,44].

Reactive astrocytes are also an important source of glial scar-related ECM. They can synthesize and secrete chondroitin sulfate proteoglycans (CSPGs) in large quantities, such as neurocan, brevican and phosphacan. CSPGs inhibit neurite growth and form a chemical inhibitory barrier around the electrode, preventing neurons from re-approximating the electrode interface [45,46,47].

Glial scars have dual biological roles. They can confine injury and limit the spread of inflammation, yet around neural electrodes their dense cellular and matrix components increase the physical distance and electrical impedance between recording sites and nearby neurons. This structural change contributes to chronic signal attenuation. Reducing mechanical mismatch and modulating ECM deposition, for example, with soft materials, hydrogels, or chondroitinase ABC to degrade CSPGs, are therefore important approaches to improving chronic interfaces [13,45,48].

### 2.5. Oxidative Stress, Neuronal Damage and Signal Attenuation

In the chronic inflammatory microenvironment, neurons around the electrode are simultaneously exposed to multiple damage factors such as oxidative stress, inflammatory factors, excitotoxicity, and mechanical micromotion. Sustained activation of microglia and macrophages can produce ROS and nitric oxide (NO). Excessive ROS can trigger lipid peroxidation, protein oxidation and DNA damage, and damage mitochondrial function, affecting neuronal energy metabolism and cellular homeostasis [2,35].

The inflammatory response may also induce excitotoxicity through an imbalance in glutamate homeostasis. For example, TNF-α can affect the uptake of glutamate by astrocytes and increase extrasynaptic glutamate levels. Excessive glutamate continuously activates NMDA receptors, leading to calcium ion influx, and further activating proteases, phospholipases, and mitochondrial damage pathways, thereby aggravating neuronal degeneration [35,49].

In addition, the relative displacement of the electrode–brain tissue caused by breathing, heartbeat, and head movement can form continuous micromechanical stimulation around the electrode. This chronic micro-motion can repeatedly perturb blood vessels and cellular structures, sustaining inflammation and tissue remodeling processes.

There is also a mutually reinforcing relationship between electrical attenuation and neuronal damage. The shrinkage of neurons increases the distance between the recording site and the signal source, causing the unit discharge amplitude to decrease; the increase in impedance increases the noise level and reduces stimulation efficiency. As stimulation threshold increases, the system may require higher charge injection to maintain the same functional output, thereby increasing the risk of electrochemical side effects and local tissue stress. Couplings between neuronal loss, glial encapsulation, and electrochemical loading may jointly drive the chronic failure process.

### 2.6. Inflammation Resolution and Tissue Repair

Inflammation should not be viewed simply as a unidirectional process of tissue destruction. Along with the initiation of inflammation, the host also engages endogenous programs for inflammatory resolution and tissue repair. For neural electrode interfaces, future material design should not only pursue broad-spectrum inhibition of inflammation, but should limit excessive inflammation while preserving necessary processes such as cell debris clearance, vascular repair, and local tissue remodeling.

Therefore, the core goal of pro-reparative neuroimmune regulation is to guide the interface response from a persistent pro-inflammatory and inhibitory scar state to a local microenvironment that is resolvable, repairable and relatively stable. This idea provides a mechanistic basis for the subsequent design of emerging immune regulatory interfaces.

In addition to cytokines, specialized pro-resolving mediators (SPMs) also provide an important mechanistic basis for interface immune regulation. SPMs are a class of lipid mediators derived from polyunsaturated fatty acids, including resolvins, protectins, and maresins. Different from extensive immunosuppression, SPMs place more emphasis on actively promoting the resolution of inflammation, including limiting neutrophil infiltration, promoting apoptotic cell clearance (efferocytosis), and inducing immune cells to switch to repair functions [50,51,52].

Single-cell sequencing studies further suggest that there are subpopulations of microglia with specific transcriptional characteristics in damage repair and neurodegenerative diseases, often called disease-associated microglia (DAMs) or repair-associated microglia. Such cells can participate in the clearance of cellular and myelin debris and may influence tissue remodeling and inflammation resolution [53,54].

In addition, regulatory T cells (Tregs) are also considered to be potentially involved in the regulation of immune homeostasis at the chronic interface. Tregs maintain immune homeostasis through secretion of IL-10, TGF-β and cell contact-dependent mechanisms. Immobilizing chemokines such as CCL22 or CCL17 on the electrode surface is expected to recruit Tregs locally and form a relatively immunomodulatory interface microenvironment. This direction provides new immunological ideas for neural electrode FBR control, but more direct verification in chronic implantation models is still needed [50].

Pro-restorative regulation provides a different idea for the design of neural electrode interfaces than broad-spectrum anti-inflammatory. Complete suppression of inflammation may impair cellular debris clearance, vascular repair, and tissue remodeling, while simply reducing proinflammatory factors may not restore neuron-electrode close coupling. A more appropriate design goal is to guide inflammation from a persistently activated state to a resolvable, repairable, and remodelable state, allowing for a more stable local homeostasis among microglia, astrocytes, and neurons.

**Figure 2:**
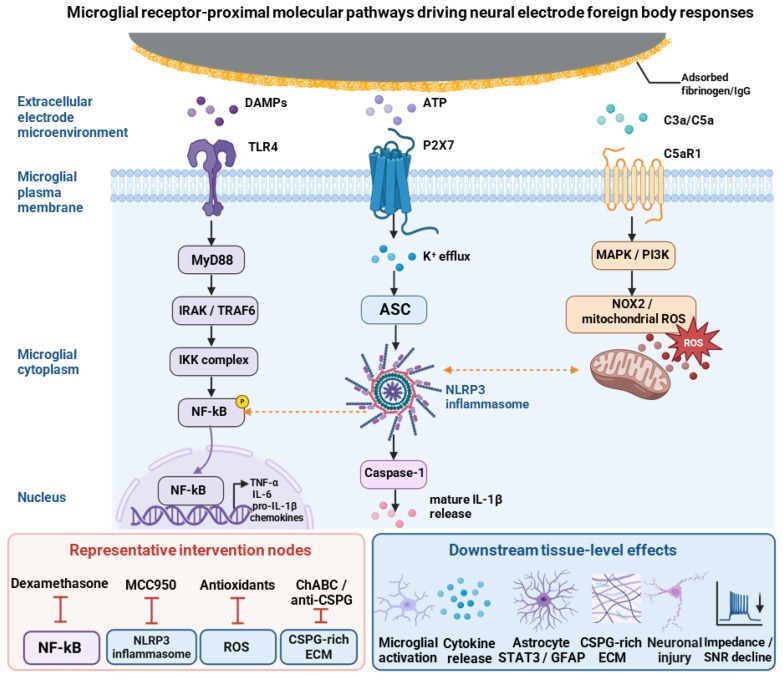
Microglial receptor-proximal molecular pathways driving neural electrode foreign body responses. Adsorbed fibrinogen, immunoglobulins, DAMPs, extracellular ATP and complement fragments activate membrane receptors including TLR4, P2X7 and C5aR1 on microglia/macrophages. TLR4-MyD88-IRAK/TRAF6-IKK signaling promotes NF-κB nuclear translocation and inflammatory gene transcription, while P2X7-driven potassium efflux promotes ASC recruitment, NLRP3 inflammasome assembly, caspase-1 activation and maturation of IL-1β. C5aR1, MAPK signaling, NOX2 activity and mitochondrial ROS further amplify inflammatory signaling. Representative intervention nodes include dexamethasone-mediated suppression of NF-κB-associated transcription, MCC950-mediated NLRP3 inhibition, antioxidant strategies targeting ROS amplification, and anti-scar approaches such as ChABC-mediated modulation of CSPG-rich extracellular matrix. Color and symbol key: purple, blue, and orange modules denote the TLR4/NF-κB, P2X7/NLRP3, and C5aR1/ROS pathway branches, respectively; black arrows indicate signaling, orange dashed arrows indicate pathway crosstalk or amplification, and red blunt-ended lines indicate inhibition at representative intervention nodes. This conceptual schematic contains no error bars.

Pro-repair pathways suggest that the design of neural electrode interfaces should not be limited to passive suppression of inflammation, but should also consider actively regulating inflammation resolution and tissue repair processes. This understanding provides a mechanistic basis for the emerging immune regulatory interfaces discussed in Section 4. The receptor-proximal microglial and intracellular signaling network is summarized in Figure 2, highlighting how receptors such as TLR4, P2X7 and C5aR1 convert protein adsorption, DAMPs, ATP and complement fragments on the electrode surface into molecular events such as NF-κB, MAPK, NLRP3 inflammasome, Caspase-1 and IL-1β.

### 2.7. Multidimensional Evaluation Metrics for Interface Responses

Accurate evaluation of FBR at the neural electrode–tissue interface and of intervention effects requires complementary electrical, histological, and molecular measures. Impedance, SNR, the number of resolvable units, and stimulation threshold reflect device function but do not identify underlying molecular mechanisms [2,14]. Histological markers such as Iba-1/CD68, GFAP, and NeuN reveal the spatial distribution of immune cells, astrocytes, and neurons. ELISA, qPCR, and proteomics can quantify key mediators, including TNF-α, IL-1β, IL-6, NLRP3, NF-κB, ROS, and IL-10. Table 1 integrates molecular events, principal cell types, and relevant evaluation metrics across implantation stages [21,44].

Current evaluation frameworks are also limited by insufficient temporal matching between electrical indicators and molecular indicators. Many studies report impedance, histological staining, and molecular expression separately at different time points, but these data are often not from the same electrode site or the same individual animal, making it difficult to establish causal relationships. Ideally, chronic implantation studies should continuously record electrical performance on the same device and perform distance-stratified histology and molecular testing at preset endpoints to determine whether a certain functionalization strategy reduces the initiation of inflammation, delays scar maturation, or mainly improves the electrode–electrolyte interface itself [21,25].

On the basis of the above indicators, the evaluation system also needs to reflect the characteristics of “partial interfaces” rather than “whole organization”. Molecular reactions in the tens to hundreds of microns around neural electrodes most directly affect recording quality, while average expression across an entire brain region may dilute key changes. For high-density electrode arrays, heterogeneity between different channels should also be considered: some sites on the same implant may maintain low impedance and high SNR, while other sites may rapidly fail due to local vascular damage, colloid wrapping, or coating defects. This site-level difference suggests that future studies should pair the electrical performance of single channels with the histological and molecular characteristics of their corresponding spatial locations whenever possible [55,56].

## 3. Material Platforms Supporting Surface-Functionalized Neural Electrodes

Surface biofunctionalization largely affects the molecular interactions between electrodes and host tissue, but the bulk material still forms the physical and chemical basis for the functionalization strategy. The bulk material determines the conductivity, mechanical compliance, chemical stability and modifiability of the electrode, and affects the initial damage, protein adsorption and coating stability after implantation. An overview of several types of representative materials from the perspective of functionalized platforms will help clarify the electrical, mechanical and chemical boundary conditions required for interfacial molecular regulation [2,14].

Bulk materials and surface biofunctionalization should not be treated as independent design layers. The substrate material determines the stiffness, deformation mode, conductive path and processability of the electrode, which in turn affects implant damage and long-term micromotion; the surface functional layer determines protein adsorption, cell recognition, drug release and local immune regulation. If the substrate is too stiff, even if the surface is equipped with an anti-inflammatory coating, long-term mechanical stimulation may continue to drive FBR; if the surface functional layer is unstable, even the most compliant electrode may lose its molecular control ability in a chronic environment. The material platform therefore forms the fundamental boundary condition [57,58] for the functionalization strategy to work.

### 3.1. Conductive Materials and Interfacial Electrochemical Functions

Electrical conductivity is the basis for neural electrodes to achieve recording and stimulation functions. Traditional metal materials such as platinum, iridium, tungsten and stainless steel have good electrical conductivity and stability, but there is a significant mismatch between their high stiffness and the soft mechanical characteristics of brain tissue. To improve electrode–electrolyte interface performance, reduce impedance, and enhance charge transport capabilities, conductive polymers and carbon-based/two-dimensional nanomaterials are widely used for surface modification of neural electrodes [1,59]. Liquid-metal conductors are another candidate platform for compliant neural interfaces because they combine high electrical conductivity with fluidic deformability; however, robust encapsulation, structural stability, and long-term biological validation remain essential [60,61,62].

In specific implementation, surface biofunctionalization also needs to consider the functional differences in different areas of the electrode. The recording site requires low impedance, stable charge exchange and close coupling of neurons; the non-recording area emphasizes antifouling, insulation and reduced cell adhesion; the flexible connection area must withstand repeated deformation without coating cracking. Functional solutions for chronic applications usually require the development of regional, hierarchical and sequential interface designs, and uniform coating is often difficult to meet all functional requirements [63,64].

Carbon-based or two-dimensional nanomaterials such as carbon nanotubes (CNTs), graphene, and MXenes provide another type of conductive interface. This type of material has a high specific surface area, good conductivity and abundant surface chemical sites. Its modification can improve the electrochemical performance of the electrode and provide an interface basis for covalently immobilizing biomolecules or loading drugs. For example, PEDOT/CNT coating can reduce impedance and alleviate local tissue damage in dorsal root ganglion stimulation model, while MXene/poly(3,4-ethylenedioxythiophene):poly(styrenesulfonate) (PEDOT:PSS) microscale fiber electrode embodies the latest trend of combining two-dimensional conductive materials with flexible microelectrode structures [65,66]. Reviews on the neural interface applications of two-dimensional nanomaterials and carbon nanomaterials in recent years also suggest that material conductivity, surface functional groups, ion exchange capabilities and long-term biological safety need to be evaluated as a whole [67,68]. However, the long-term migration, degradation behavior, potential neurotoxicity, and batch-to-batch consistency of nanomaterials still need to be further evaluated in chronic implantation models [69].

The value of conductive material platforms is not limited to reducing impedance but also providing functionalized interfaces that can be coupled with drug delivery, ligand immobilization, and responsive coatings. The evaluation of conductive materials in the field of neural electrodes should not stop at the conductivity or impedance value itself, but should further analyze how electrochemical improvements change local charge transport, protein adsorption, inflammatory mediator generation, drug release, and neuron-electrode coupling. PEDOT:PSS-based soft and stretchable bioelectronics further illustrate how a conducting polymer can be engineered to retain electrical function while improving mechanical compatibility with tissue [70].

### 3.2. Mechanical Adaptation Strategies for Soft Materials and Flexible Electrodes

Brain tissue is a low-modulus soft tissue, and the modulus of conventional silicon-based or metal microelectrodes is often orders of magnitude higher. This mechanical mismatch will continue to generate local stress in the micromotions caused by breathing, pulse and head movement, and is an important physical cause of chronic FBR [3]. The design goal of flexible and soft material electrodes is to reduce long-term mechanical stimulation while maintaining electrical function [71,72]. In addition to the ultra-flexible electronic probe [73,74], ultrasoft microwire electrodes have also shown potential to improve chronic tissue integration, suggesting that lowering bending stiffness and reducing fretting stress can directly affect glial wrapping, neuronal distance and long-term interface stability [75].

Ultra-small, ultraflexible nanoelectronic and mesh probes developed in the laboratory of C. M. Lieber provide an important counterpoint to the substantial tissue responses commonly observed around conventional neural electrodes. Studies of these tissue-like devices reported little or minimal chronic immune response, markedly reduced glial encapsulation, and stable single-unit recording after implantation in the rodent brain. In particular, syringe-injectable mesh electronics enabled stable mapping at the single-neuron level over several months, whereas scalable mesh probes maintained chronic multichannel recordings while preserving an ultraflexible, macroporous architecture. These observations suggest that the canonical progression from implantation injury to chronic signal deterioration is not invariant across electrode designs [71,76,77,78,79].

These results should not, however, be interpreted as evidence that foreign body responses are universally absent in ultraflexible probes. Rather, they indicate that the magnitude and detectability of the response are strongly design- and implantation-dependent. Submicrometer-scale thickness, low bending stiffness, an open mesh geometry, and syringe-assisted deployment may collectively reduce tissue displacement and persistent micromotion, thereby changing the local conditions that normally sustain glial activation and scar remodeling. Direct comparison across device geometries, brain regions, implantation routes, observation periods, and histological or electrophysiological endpoints remains necessary before these findings can be generalized to other chronic neural interfaces [71,78].

Hydrogels provide an interfacial environment that more closely resembles soft tissue. As three-dimensional polymer networks with high water content, they have low moduli and can reproduce selected extracellular-matrix characteristics [75,80]. Incorporating conductive polymers or nanomaterials into hydrogel matrices, such as PEG, hyaluronic acid, and chitosan, can combine electrical conductivity with mechanical compliance. Hydrogels can also serve as reservoirs or release platforms for anti-inflammatory drugs, neurotrophic factors, and ECM-mimetic ligands [59]. Conductive hydrogel electrodes have been used for conformal brain-surface recording, where low impedance and improved tissue compatibility were demonstrated; hydration control, swelling, and long-term durability nevertheless remain important limitations [81,82,83].

Soft materials and flexible platforms mainly improve the interface state by reducing sustained mechanical stimulation after implantation and providing a relatively mild matrix environment for the local integration of biofunctional molecules. Key challenges include mechanical support during implantation, long-term packaging reliability, wire connection stability, and the balance between conductivity and compliance.

The development of flexible electrodes also suggests that geometry is as important as material modulus. Elongated, thin-layered, mesh-like or fibrous structures can significantly reduce the effective bending stiffness and reduce the local stress concentration caused by relative motion even if the modulus of the material itself is not completely close to that of brain tissue. However, overly soft devices may face challenges such as difficult implantation, unstable positioning, fragile packaging, and complex wire interconnections. Thus, optimization of soft material platforms should strike a balance between implant maneuverability, long-term mechanical stability, and interfacial biocompatibility.

### 3.3. Co-Design Principles for Material Platforms and Surface Biofunctionalization

Whether it is a conductive platform or a flexible platform, modern neural electrode design is moving from single material performance optimization to systemic interface engineering. The bulk material provides electrical stability, mechanical compliance, and a modifiable surface; the surface functional coating directly participates in molecular events such as protein adsorption, immune recognition, cell adhesion, and drug release, and determines the regulation direction of the interface microenvironment.

A platforming perspective helps avoid overreliance on a single representative material. The impact of PEDOT, CNT, MXene, hydrogels and flexible polymers on long-term stability is mainly due to the combined effects of multiple factors such as charge transport, mechanical coupling, surface chemistry and molecular delivery. Promising neural electrode materials should be composable, i.e., be spatially compatible with antifouling, pro-neuronal, anti-inflammatory, and pro-repair modules, and maintain structural and functional integrity during long-term implantation [57,84].

## 4. Surface Biofunctionalization Strategies Targeting Molecular Pathways

**Figure 3:**
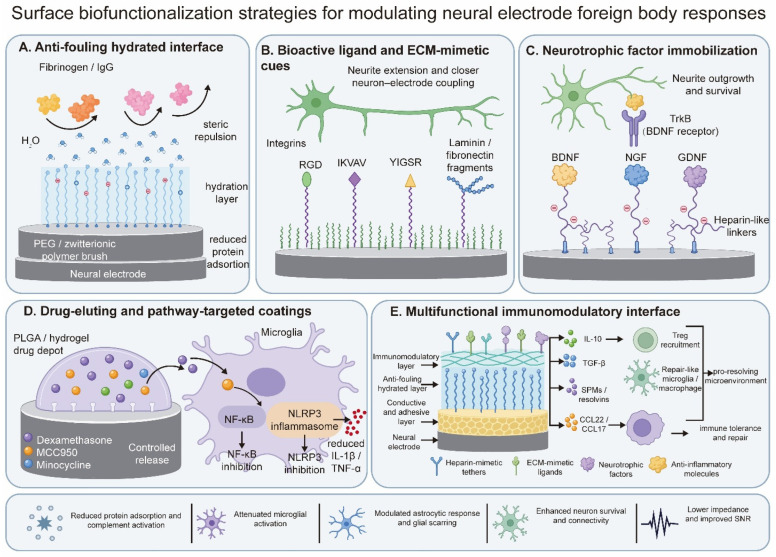
Surface biofunctionalization strategies for neural electrodes. (**A**) Antifouling barriers reduce nonspecific protein adsorption and early immune recognition. (**B**) Bioactive ligand and extracellular-matrix cues promote cell-selective integration and neuronal contact. (**C**) Neurotrophic factor immobilization provides localized survival and growth signals. (**D**) Drug-eluting and responsive coatings suppress inflammatory and scar-forming cascades. (**E**) Multifunctional immunomodulatory interfaces combine soft mechanics, spatial patterning and pro-resolution signaling for chronic implants. Color and symbol key: colored motifs distinguish the five functionalization strategies and their associated surface components or molecular and cellular targets; arrows indicate intended biological effects, and red blunt-ended lines indicate inhibition of the indicated pathway.

Surface biofunctionalization is a key step in converting molecular mechanisms into interface regulation strategies. The main molecular events surrounding FBR include antifouling coatings, bioactive ligands, neurotrophic factor immobilization, drug delivery, and emerging immune regulatory interfaces, which respectively act on protein adsorption, immune recognition, glial response, neuronal survival, and tissue repair processes. Drawing on prior reviews [2,14], these strategies and their main sites of action are summarized in Figure 3, which provides a graphical index for the major surface biofunctionalization pathways. A comparative overview is provided in Table 2.

From a molecularly informed perspective, the choice of surface-biofunctionalization strategy should match the dominant stage of FBR. Antifouling and complement-regulating approaches are most relevant to initial immune recognition. ECM-like ligands, neurotrophic factors, and spatial patterning are intended to preserve close neuronal coupling. Drug delivery and responsive coatings are more directly targeted at subacute inflammatory amplification and scar formation. Combination strategies require careful consideration of their timing, spatial placement, and long-term stability [85,86].

Building on the mechanistic basis outlined in Section 2, the following subsections assess surface-functionalization strategies according to the stage of FBR targeted, the evidence available in neural-electrode or related implantation models, and their principal technical limitations.

### 4.1. Antifouling Coatings: Regulation of Initial Protein Adsorption and Immune Recognition

Antifouling coatings primarily target nonspecific protein adsorption, fibrinogen-mediated immune recognition, complement activation, and early innate immune responses. Because FBR begins with rapid protein adsorption on the electrode surface, low-fouling coatings are designed to create a stable hydration layer or otherwise low-interaction interface that limits protein and cell adhesion, complement deposition, immune recognition, and early inflammatory activation [19,84].

PEG/PEG-like polymers and zwitterionic coatings are the most commonly discussed antifouling systems. High-density PEG chains can form a hydration layer on the surface that binds water molecules and has chain segment mobility. When proteins approach this interface, they need to destroy the hydration layer and compress the polymer chains, so it is thermodynamically unfavorable for stable adsorption. Zwitterionic polymers (such as poly(sulfobetaine methacrylate), PSBMA) side chains contain both positive and negative charged groups, which can form a stable hydration shell through strong ionic hydration, and usually exhibit strong anti-protein adsorption capacity and good antioxidant stability [84].

Heparinized or heparin-like coatings may provide both antifouling and immunomodulatory effects. Heparin is rich in sulfate and carboxyl groups, giving it a high negative charge density and strong hydration capacity [84,85]. Surface-immobilized heparin can reduce protein adsorption and may attenuate early inflammatory responses by modulating coagulation- and complement-related processes. At neural-electrode interfaces involving microvascular injury and plasma-protein extravasation, heparinoid surfaces are therefore mechanistically plausible [2,87,88].

Although antifouling coatings can attenuate early FBR, long-term stability and functional selectivity remain important limitations. Polymers such as PEG may degrade in oxidative or hydrolytic physiological environments, and coatings can delaminate from their substrates [59,86]. Antifouling design should not seek to eliminate all cell contact at recording sites. Rather, it should preserve close neuronal coupling near recording sites while limiting glial adhesion over the overall implant surface. A spatially partitioned design may therefore be preferable, with low-fouling regions in non-recording areas and controlled neuron-interactive regions near recording sites [14,89,90,91,92].

The difficulty of antifouling strategies lies in the tension between “low reactivity” and “functional coupling” [14,84]. An ideal stain-resistant surface can reduce fibrinogen adsorption, complement deposition, and immune cell adhesion, but the neural recording site needs to be electrically close enough to surrounding neurons. An overly inert interface may reduce inflammation while simultaneously impairing neuronal adhesion and neurite proximity. Future antifouling designs are more likely to adopt regionalized or gradient schemes to minimize biological contamination in non-recording areas and preserve controllable neuronal interactions near the recording site [93,94]. Direct neural-implant evidence currently supports an acute reduction in microglial surface encapsulation by PSBMA, whereas chronic electrophysiological benefit remains to be established [90].

### 4.2. Bioactive Ligands and ECM Fragment Modifications

Bioactive ligands and extracellular-matrix fragments are intended to preserve neuronal proximity during the subacute-to-chronic phase by providing cell-instructive surface cues. Their effectiveness depends less on re-explaining integrin signaling than on ligand density, orientation, spatial presentation, and selectivity for neurons over reactive glia [14,59,95,96,97].

The key to this strategy is not to increase the number of bioactive molecules, but to control their density, orientation, spatial arrangement, and linker length. Improper ligand presentation may fail to improve neuronal selectivity or even promote astrocyte or microglia adhesion. Therefore, optimizing ligand density, conformational exposure, and compartmentalized distribution is key to improving the effectiveness of this strategy. Embedding pro-adhesion ligands into an antifouling background may help balance low inflammatory responses with close neuronal coupling [96,97].

The focus of ligand modification is to improve cell selectivity and signal directivity rather than simply enhancing adhesion strength. Broad-spectrum adhesion sequences such as RGD may promote the attachment of multiple types of cells at the same time. If there is a lack of spatial control, they may enhance glial cell coverage. In contrast, specific peptides derived from laminin or the neurodevelopmental microenvironment are more suitable for promoting neurite growth and neuronal proximity. Ligand density, spacing, flexible linker length, and background antifouling layer collectively determine receptor aggregation and downstream focal adhesion signaling, so this strategy requires precise interface presentation rather than simply increasing molecule loading.

### 4.3. Immobilization of Neurotrophic Factors

Neurotrophic factor immobilization mainly targets the Trk receptor family, p75NTR receptor, and downstream pro-survival and growth-promoting pathways such as PI3K/Akt and MAPK/ERK. Neurotrophic factors support neuronal survival, differentiation, and neurite growth. In the inflammatory and oxidative stress microenvironment formed by electrode FBR, local presentation of neurotrophic factors can activate neuronal pro-survival pathways, alleviate degenerative changes, and maintain functional signal sources near the electrode [2,59].

Conductive polymers, particularly PEDOT:PSS and polypyrrole (PPy), can substantially reduce electrode–electrolyte interfacial impedance because of their mixed ionic and electronic conductivity [1]. Depending on their preparation, these coatings can develop rough or porous microstructures that increase effective surface area and support drug loading, biomolecule immobilization, and electrically triggered release [98,99]. Composite coatings, including PEDOT/MWCNT, PEDOT/CNT, and PEDOT–ionic-liquid systems, have been evaluated for electrochemical performance, chronic recording stability, and recording or stimulation function in vivo [100,101,102]. Conductive polymers should therefore be viewed not only as impedance-reducing electrochemical layers but also as functional platforms for carbon nanomaterials, anti-inflammatory drugs, and electrically responsive release systems. By lowering impedance and increasing charge-injection capacity, they may reduce local charge density and limit water electrolysis, pH shifts, and Faradaic reactions during strong polarization. Reduced ROS generation, acid–base perturbation, and electrochemical by-products may in turn lessen neuronal membrane injury, glial activation, and oxidative-stress amplification during stimulation [103,104]. The performance of PEDOT:PSS-based bioelectronics depends on balancing conductivity with softness and stretchability, rather than optimizing either property in isolation [70].

The advantage of neurotrophic factor immobilization is that it can directly support the survival of neurons near the electrode and stabilize the signal source. Its main limitations include unstable protein conformation, possible loss of activity due to the immobilization process, limited half-life in vivo, and high cost. Combining immobilized delivery with a controlled release system may help to extend its local action time [59,105].

The advantage of the neurotrophic factor strategy is that it directly targets the signal source cells, but its effect is highly dependent on local spatiotemporal presentation. Freely diffusing factors may be rapidly diluted or degraded, while excessively strong or prolonged growth-promoting signals may cause non-target cell responses or abnormal neurite growth. Immobilized presentation can increase local concentration and reduce systemic exposure, but the immobilization process may alter protein conformation and receptor accessibility. Therefore, neurotrophic factors are more suitable as part of composite interfaces, used in conjunction with a low inflammatory background, an appropriate mechanical platform, and a stable electrochemical interface. At present, this rationale is supported mainly by neural-repair and interface studies, with comparatively limited long-term validation in chronically recording neural electrodes [2,59,105].

### 4.4. Drug Delivery Electrodes: Local Regulation of Pro-Inflammatory and Anti-Scar Pathways

Drug-delivery electrodes target pro-inflammatory and pro-scarring mediators, including NF-κB, MAPK, NLRP3, TNF-α, IL-1β, ROS, and CSPGs. They release small molecules, proteins, or immunomodulators locally from the electrode body or surface coating, thereby restricting drug exposure spatially and aligning treatment with FBR stages. Local delivery may increase interfacial drug concentrations while reducing systemic adverse effects relative to systemic administration [2,106]. Conducting-polymer films can also use electrochemical doping and dedoping to achieve electrically controlled neurochemical release, converting passive recording or stimulation devices into locally regulated interfaces with temporal control [107].

The evidentiary basis differs substantially among these targets. Controlled dexamethasone release has been evaluated directly in a 12-week neural-microelectrode implantation study, with stable recording and impedance and closer neuronal proximity at actively released sites [108]. In contrast, the rationale for NLRP3 inhibition remains largely mechanistic in the neural-electrode setting, and the principal functional evidence for ChABC-mediated CSPG degradation derives from spinal cord injury rather than chronic electrode implantation [45]. These approaches should therefore not be assigned equivalent levels of evidence.

Hydrogels can embed drugs through network pores and release them through diffusion, swelling or degradation. Their soft mechanical characteristics also make them suitable as an interface layer with both mechanical buffering and drug delivery functions [59,99]. Conductive polymers can introduce charged drug molecules into the network as doping ions during the electropolymerization process, and then realize ion exchange and drug release through potential regulation, giving the electrode itself the potential for electrical response or on-demand release. pH-, ROS- or enzyme-responsive polymers can also release drugs based on changes in the inflammatory microenvironment, such as triggering drug release under local acidification or elevated ROS conditions, thereby matching drug release to pathological conditions [109,110]. Electrochemically integrated conducting-polymer/hydrogel architectures offer an additional route to combine a compliant tissue-facing layer with passive or electrically triggered cargo release [111].

Drug-delivery design should be matched to the temporal course of FBR. Acute interventions aim to limit early inflammatory activation, whereas subacute approaches target glial scar formation and chronic strategies may require sustained low-dose neurotrophic or immunomodulatory support. For stimulation electrodes, residual voltage after biphasic stimulation can indicate electrode damage or irreversible electrochemical reactions. Drug-release and conductive-coating evaluations should therefore consider the electrochemical safety window as well as histological endpoints [49,112]. Current limitations include burst release, limited drug loading, short release duration, and poorly controlled release profiles. Drug-eluting electrodes also face a spatiotemporal mismatch: degradable carriers and hydrogel coatings commonly release cargo for days to months, whereas clinical interfaces may need to function for years. Moreover, regions of highest drug concentration may not coincide with the microdomains of chronic inflammation, scar remodelling, and neuronal loss. Drug-eluting electrodes are thus best viewed as strategies for regulating early and subacute interface responses, rather than as stand-alone solutions for lifetime device failure [106,113,114,115].

Drug-delivery strategies also face regulatory and manufacturing challenges during clinical translation. Combining drugs, electrodes, and coatings creates a device–drug combination product rather than a conventional medical device. Batch consistency, release kinetics, sterilization effects, long-term residues, and failure modes all require systematic evaluation. In research prototypes, short-term release may establish biological activity. For chronically implanted devices, however, the more important question is whether the interface retains an acceptable tissue response after drug depletion and whether carrier degradation introduces new inflammation or electrochemical instability [116,117].

The endpoints of neural electrode interface studies should not be limited to demonstrating that a material reduces a certain inflammatory marker at a single time point. More convincing evidence should demonstrate that the interface is capable of maintaining interpretable, reproducible, and translatable tissue-device interaction states under chronic electrical operating conditions. This judgment standard requires researchers to simultaneously pay attention to local molecular networks, cellular spatial distribution, electrochemical safety windows, and long-term functional output, and gradually advance short-term mechanism verification to systematic evidence sufficient to support chronic applications.

Rather than considering bulk materials and surface functionalization independently, neural-electrode design should account for their interaction. Tissue-like and mesh neural probes illustrate how device architecture and interfacial design can jointly influence tissue integration [118,119].

### 4.5. Emerging Immunomodulatory Interfaces: Opportunities and Evidence Gaps

The emerging multifunctional and immune regulatory interface mainly involves IL-10, TGF-β, SPM receptors, FOXP3, integrin network and repair-related cytokine network [40,56]. Compared with traditional anti-inflammatory strategies, such strategies emphasize inducing local immune responses to shift toward inflammation resolution, tissue repair, and immune tolerance, rather than just broadly suppressing inflammation intensity. This idea is closer to the biological state required for long-term stability of the chronic implant interface [50,51].

Although this design rationale is conceptually attractive, the evidence supporting IL-10 delivery, specialized pro-resolving mediators (SPMs), and CCL22-mediated Treg recruitment should be interpreted cautiously. Their pro-resolution or immune-homeostatic rationale is supported primarily by inflammatory disease, CNS injury, or non-neural biomaterial models; direct chronic neural-electrode validation remains limited [51,52,54].

For neural interfaces, the critical unresolved questions concern delivery stability, dose and effective time window, spatial restriction to the electrode–tissue interface, and long-term effects on immune surveillance. Future studies should therefore combine molecular and histological endpoints with longitudinal electrophysiology and should test whether benefits persist after the delivered signal is depleted [50,51,52,56].

**Table 2:** Surface biofunctionalization strategies and their molecular targets.

Strategy	Representative Modality	Target/Intended Outcome	Evidence Strength	Main Effective Window	Key Limitation	References
Antifouling barrier	PEG, zwitterionic polymer, heparin	Protein adsorption/early immune recognition	Direct acute neural-implant evidence	Acute	Durability; may reduce neuronal coupling	[84,86,90]
Bioactive ligand	RGD, IKVAV, YIGSR, laminin	Cell-selective adhesion/neurite proximity	Preclinical neural-interface evidence	Subacute-chronic	Ligand presentation; possible glial adhesion	[95,96,97]
Neurotrophic presentation	Immobilized BDNF, NGF, GDNF	Neuronal survival/local trophic support	Related neural-repair evidence; limited chronic electrode validation	Subacute-chronic	Bioactivity and release duration	[59,105]
Drug-eluting interface	Dex, minocycline, IL-1Ra, ChABC	Inflammation or scar modulation	Direct chronic evidence for selected agents; target-specific evidence varies	Acute-subacute	Burst release; finite duration	[106,108,113,114,115,116,117]
Immunomodulatory interface	IL-10, SPMs, CCL22/Treg	Resolution/immune homeostasis	Predominantly extrapolated evidence	Chronic (hypothesized)	Delivery specificity; long-term safety	[50,51,52,54,55,56]

## 5. Challenges and Prospects

Molecular-mechanism-guided surface biofunctionalization offers a coherent route to improving the long-term stability of neural electrodes. Its translation from laboratory prototypes to reliable clinical products remains constrained by material limitations, the complexity of the chronic implant environment, and shortcomings in current evaluation frameworks.

### 5.1. Main Limiting Factors for Long-Term Interface Stability

Long-term stability is a common challenge across functionalization strategies [3,99]. Antifouling layers, immobilized biological ligands, and drug carriers can undergo hydrolysis, oxidation, enzymatic degradation, or mechanical wear in vivo. Adhesion between a coating and its electrode substrate can also decline over time, causing delamination or local functional failure [71,76,113].

For drug delivery systems, the effective release time of degradable polymers or hydrogels is usually weeks to months, which is difficult to cover the years-long use requirements of chronic neural interfaces [49,106]. Achieving long-term, stable, low-dose local release is still a key technical issue facing drug-eluting electrodes. At the same time, conductive polymers may undergo peroxidation or structural rearrangement and lead to a decrease in electrical properties, hydrogels may swell, dehydrate, or degrade, and nanomaterials may suffer from long-term migration, degradation, and safety uncertainties. These factors all affect the overall longevity of the functionalized interface [59,116].

### 5.2. Trade-Off Between Mechanical Compliance and Multifunctional Integration

Flexible neural electrodes are commonly fabricated from polymers such as polyimide (PI), polyparaxylene (Parylene-C), and polydimethylsiloxane (PDMS). Relative to rigid microelectrode materials such as silicon and metals, these polymers can have lower effective stiffness or be processed into thin, flexible films. The bulk moduli of PI and Parylene-C nevertheless remain substantially higher than that of brain tissue. Their mechanical advantage therefore arises not only from material modulus but also from reduced thickness, microstructural design, and lower overall bending stiffness [120,121].

Good mechanical compliance is an important physical factor in mitigating FBR, but complex functionalization may conflict with compliance [3,71]. Superimposing an adhesive layer, drug layer and protective layer on an ultra-flexible substrate may increase the thickness and effective stiffness of the device, thus weakening the advantages of the flexible substrate. Multilayer structures may also introduce problems such as interlayer delamination, interface fatigue, and unstable release behavior. Therefore, how to integrate molecular regulatory functions while maintaining mechanical matching is a core trade-off in neural electrode interface engineering [72,120].

Long-term interface stability should be distinguished from short-term coating efficacy. Many coatings reduce inflammatory markers or improve impedance soon after implantation, yet their chemical integrity, mechanical adhesion, and bioactivity may decline within weeks. If a functional layer fails and exposes a rougher or more protein-adsorptive substrate, it may provoke secondary inflammation. Evaluations of functionalized electrodes should therefore report coating integrity, drug residues, surface chemistry, and electrical properties alongside endpoint histology.

### 5.3. Limitations of In Vivo Validation Standardization and Molecular Evaluation Systems

Currently, no consensus evaluation framework has been established for assessing neural electrode biocompatibility and long-term interface stability [2,14]. There are significant differences between different studies in animal models, implantation sites, implantation times, recording parameters, and histological analysis methods, making cross-sectional comparison of results difficult. Although general biocompatibility evaluation frameworks can provide a basic reference, specialized evaluation systems for the long-term functional stability and interface molecular reactions of chronic neural implants still need to be improved [15,21].

Many studies have reported improvements at the histological or electrophysiological level with functionalized electrodes, such as reducing glial scar thickness or improving SNR. However, whether these improvements are indeed caused by the regulation of target molecular pathways still requires more direct mechanistic evidence. Future studies need to simultaneously evaluate electrode performance, cellular responses, and the expression or activation status of key pathway molecules during acute, subacute, and chronic stages to establish clearer causal relationships. Single-cell RNA sequencing (scRNA-seq), spatial transcriptomics, and multiplex imaging techniques (such as CODEX) can resolve different cell subpopulations and their spatial distribution at the electrode interface at higher resolutions. Combining these tools with electrophysiological and histological indicators can help identify novel FBR regulatory nodes and evaluate the true mechanism of action of functionalization strategies [21,25].

The absence of standardized evaluation also limits comparison across material strategies. Studies differ in electrode size, implantation depth, animal strain, recording algorithm, tissue-section distance, and staining quantification, making claims such as reduced glial scarring or improved SNR difficult to compare directly. The field would benefit from minimum reporting standards that include electrode geometry, implantation-injury control, the number of recording sites, definitions of channel failure, distance-stratified histology, key molecular markers, and statistical methods. Statistical designs should also account for animal-level and within-animal correlations. Multiple electrode channels in a single animal share the same biological background, implantation environment, and postoperative response and are not independent observations. Treating all channels as independent inflates the effective sample size, underestimates standard errors, and can overstate statistical significance. More robust analyses use multilevel models at the animal, implant, and channel levels, or include animals and implants as random effects. Exclusion criteria, the proportion of failed channels, and blinded quantification procedures should be reported clearly. Transparent evaluation frameworks will enable molecularly guided interface design to accumulate comparable evidence [21,44,113,122,123,124].

**Figure 4:**
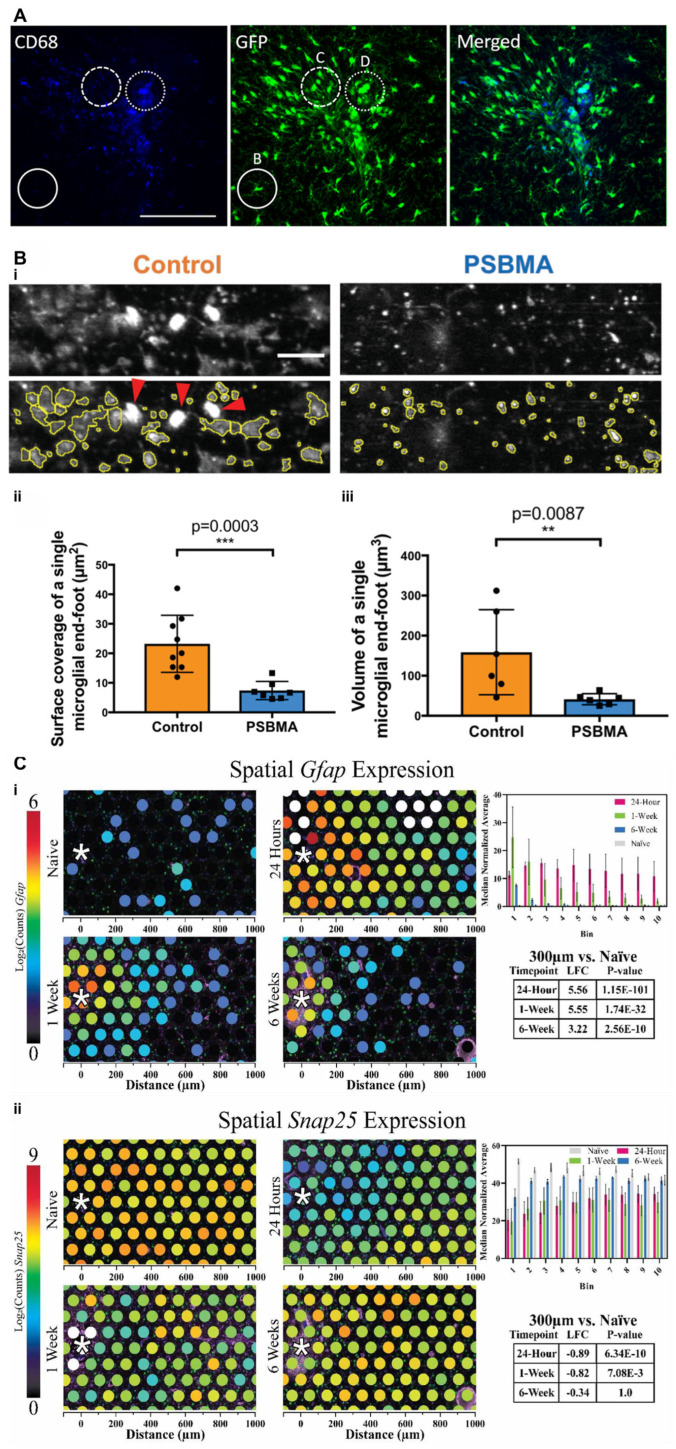
Experimental evidence linking inflammatory tissue responses, surface biofunctionalization, and spatial molecular profiling at neural electrode–tissue interfaces. (**A**) Representative CD68 and GFP immunofluorescence images showing microglia/macrophage responses around intracortical microelectrodes, with the merged image illustrating the spatial relationship between CD68-positive activated cells and GFP-positive cells near the implant site [86]. (**B**) Zwitterionic PSBMA coating decreases microglial end-foot spreading on the probe surface. (**i**) Microglial end-foot identification on control and PSBMA-coated probe surfaces at 6 h post-implantation. (**ii**) Surface coverage area of individual microglial end-feet. (**iii**) Volume of individual microglial end-feet. Together, these measurements show reduced end-foot spreading around PSBMA-coated implants [90]. Statistical annotations and *p* values are reproduced from the original study [90]. (**C**) Spatial gene-expression data around implanted electrodes. (**i**) Spatial expression of *Gfap*. (**ii**) Spatial expression of *Snap25*, indicating astroglial reactivity and neuronal-marker reduction after device implantation [21]. Symbol key: (**A**) solid, dashed, and dotted circles identify non-activated ramified microglia, activated microglia extending processes towards the implant, and highly activated microglia/macrophages, respectively; scale bars denote spatial scale. (**B**) Red arrowheads identify excluded microglial cell bodies and yellow contours delineate end-feet selected for area measurement; circles and squares identify individual control and PSBMA probe-shank measurements, respectively; bars and error bars show mean ± SD. ** *p* < 0.01; *** *p* < 0.001. (**C**) Colored circles are spatial-transcriptomic spots and their color represents log2(normalized counts); white asterisks mark the electrode site; MNA, median-normalized average; LFC, log2 fold change; naïve, non-implanted control tissue. Error bars reproduce the original variability estimates. In (**A**), the scale bar represents 100 μm. In (**B**), the white scale bar is retained from the original source; its numerical length was not reported in the original figure legend.

Representative experimental studies further illustrate the value of complementary histological, interfacial, and spatial molecular endpoints for evaluating chronic neural electrode–tissue responses. In Figure 4, CD68/GFP immunofluorescence visualizes inflammatory-cell responses around intracortical microelectrodes (Figure 4A); a PSBMA coating reduces microglial endfoot spreading and volume at the probe surface (Figure 4B); and spatial profiling of *Gfap* and *Snap25* links astroglial activation and reduced neuronal-marker expression to the local implant microenvironment (Figure 4C).

### 5.4. Molecular-Mechanism-Guided Interface Design

In the future, neural electrode design will further shift from “material-centered” to “molecular target and interface microenvironment-centered”. Responsive coatings that can sense local inflammatory factors, pH or ROS changes and release drugs on demand can improve the timing accuracy of intervention and reduce unnecessary drug exposure. Artificial intelligence and high-throughput screening technologies can be used to predict material-protein interactions, optimize surface chemical composition, and screen delivery formulations, but their results still need to be verified through in vivo chronic implantation models. Neural electrodes can also learn from nerve repair and regeneration interface strategies to integrate biological signals on the electrode surface that contribute to neuronal survival, process maintenance, blood vessel stability, or local tissue remodeling to improve long-term tissue integration without sacrificing recording or stimulation functions [125]. This development direction does not mean converting neural electrodes into tissue engineering scaffolds, but requires that the electrode interface simultaneously meets electrochemical stability, mechanical compliance, molecular regulation, and chronic safety. Future research should pay more attention to the synergistic relationship between functionalization strategies, such as spatial partitioning and temporal integration of low-impedance conductive layers, antifouling backgrounds, localized anti-inflammatory release, pro-neuronal ligands, and long-term maintenance of immunomodulatory signals [119,126]. Active biointegrated architectures, including electrolyte-engineered organic electrochemical transistors and neuromorphic processing systems, may eventually complement conventional electrodes through local signal amplification and on-device processing; their chronic interface performance, however, still requires rigorous in vivo validation [127,128].

Facing the future, neural electrode interface design may gradually move from static coatings to dynamic response systems. The ideal interface can inhibit protein adsorption and complement activation in the early stages of implantation, release anti-inflammatory or anti-scarring factors in the subacute stage, and maintain low impedance, low micromotion stress, and a pro-neuronal microenvironment in the chronic stage. Achieving this goal will require closer collaboration between materials chemistry, electrochemistry, immunology and neuroengineering, as well as validation on longer time scales and higher resolution in animal models [125,129].

## 6. Conclusions

Performance degradation of chronically implanted neural electrodes results from a continuously evolving foreign body response at the electrode–tissue interface. This process is jointly determined by device mechanical properties, electrochemical stability, and host molecular responses, and manifests as a continuous cascade from implantation damage, protein adsorption, and complement activation, to microglial pro-inflammatory signaling, astrocyte reactive proliferation, glial scarring, oxidative stress, and neuronal damage. The resulting inhibitory interface microenvironment is an important basis for chronic impedance increase and signal attenuation.

Future neural-electrode design should move beyond single-material optimization towards multiscale integration. Soft mechanical platforms can reduce implantation injury and chronic micromotion, stable electrochemical interfaces can preserve recording and stimulation performance, molecularly targeted functional layers can modulate local immunity and neuronal responses, and standardized in vivo evaluation can test long-term effects. Progress will depend on establishing closer links among materials, device design, and molecular mechanisms at the interface.

Long-term stability of neural electrodes is difficult to achieve by relying on a single material strategy. A more feasible path is to establish multi-scale design principles that can explain and regulate interface responses. The material’s flexibility, electrochemical stability, protein adsorption behavior, immune recognition method, degree of glial scar, and spatial distribution of neurons jointly affect the final recording or stimulation performance. Organizing these factors along the lines of molecular mechanisms can move surface biofunctionalization from empirical coating optimization to verifiable, comparable, and iterable interface engineering strategies.

## Figures and Tables

**Table 1:** Molecular events and evaluation metrics of neural electrode foreign body response [15,16].

Stage	Major Molecular Events	Major Cell Types	Representative Markers	Interface Consequences	Evaluation Metrics	References
Acute phase (hours-days)	Implantation damage, BBB disruption, protein adsorption, complement activation (C3b, C5a)	neutrophils, microglia	Iba-1, MPO, C3b deposition, C5a concentration	acute inflammation, cell loss	Electrophysiology (transient drop in impedance), histology (cell infiltration), molecular (complement fragments C3b/C5a, serum protein extravasation)	[16,17,18]
Subacute phase (days-weeks)	Sustained activation of microglia/macrophages (TLR/NF-κB, NLRP3), astrocyte response (JAK/STAT3)	microglia, macrophages, astrocytes	Iba-1/CD68, GFAP, p-NF-κB, NLRP3, IL-1β, TNF-α	Chronic inflammation is established and glial scars begin to form	Electrophysiology (impedance starts to rise, SNR falls), histology (glial cell layer thickness), molecular (cytokine levels)	[27,37,42]
Chronic phase (weeks-months/years)	ECM (CSPGs) deposition, oxidative stress, neuronal loss, insufficient pro-repair signaling (IL-10)	astrocytes, microglia, fibroblasts	GFAP, Vimentin, Neurocan, NeuN, MAP2, ROS markers	Mature glial scar, neuronal degeneration, interface insulation	Electrophysiology (impedance stabilizes at high level, SNR remains low), histology (scar thickness, neuron density), transcriptomics (C3, MMP12, SPP1) [7]	[11,12,20]

## Data Availability

No new data were created or analyzed in this study. Data sharing is not applicable to this article.

## Abbreviations

The following abbreviations are used in this manuscript:

FBR	foreign body response
DBS	deep brain stimulation
ECoG	electrocorticography
SNR	signal-to-noise ratio
BBB	blood–brain barrier
ECM	extracellular matrix
TLR	Toll-like receptor
NLRP3	NOD-, LRR- and pyrin domain-containing protein 3
STAT3	signal transducer and activator of transcription 3
CSPG	chondroitin sulfate proteoglycan
DAMP	damage-associated molecular pattern
ATP	adenosine triphosphate
HMGB1	high-mobility group box 1
Mac-1	macrophage-1 antigen
CD11b	cluster of differentiation 11b
CD18	cluster of differentiation 18
C1q	complement component 1q
C3	complement component 3
C3bBb	alternative-pathway C3 convertase
C3a	complement component 3a
C3b	complement component 3b
C5a	complement component 5a
C3aR	C3a receptor
C5aR1	C5a receptor 1
CNS	central nervous system
M1/M2	classically activated/alternatively activated microglial or macrophage phenotypes
ROS	reactive oxygen species
MAPK	mitogen-activated protein kinase
PRR	pattern recognition receptor
PAMP	pathogen-associated molecular pattern
TLR2	Toll-like receptor 2
TLR4	Toll-like receptor 4
MyD88	myeloid differentiation primary response 88
NF-κB	nuclear factor-κB
IL	interleukin
TNF-α	tumor necrosis factor-α
IL-1β	interleukin-1β
IL-6	interleukin-6
P2X7	P2X purinoceptor 7
ASC	apoptosis-associated speck-like protein containing a CARD
MPO	myeloperoxidase
Caspase-1	cysteine-aspartic protease 1
IL-18	interleukin-18
NADPH	nicotinamide adenine dinucleotide phosphate
DNA	deoxyribonucleic acid
PVM	perivascular macrophage
GFAP	glial fibrillary acidic protein
Iba-1	ionized calcium-binding adapter molecule 1
JAK	Janus kinase
NeuN	neuronal nuclei
MAP2	microtubule-associated protein 2
NO	nitric oxide
NMDA	N-methyl-D-aspartate
IL-10	interleukin-10
TGF-β	transforming growth factor-β
CCL22	C-C motif chemokine ligand 22
CCL17	C-C motif chemokine ligand 17
Treg	regulatory T cell
IRAK	interleukin-1 receptor-associated kinase
TRAF6	TNF receptor-associated factor 6
IKK	IκB kinase
NOX2	NADPH oxidase 2
MCC950	NLRP3 inflammasome inhibitor MCC950
ChABC	chondroitinase ABC
CD68	cluster of differentiation 68
ELISA	enzyme-linked immunosorbent assay
qPCR	quantitative polymerase chain reaction
PEDOT	poly(3,4-ethylenedioxythiophene)
CNT	carbon nanotube
MXene	two-dimensional transition-metal carbide, nitride or carbonitride
PSS	poly(styrenesulfonate)
PEG	poly(ethylene glycol)
PSBMA	poly(sulfobetaine methacrylate)
RGD	Arg-Gly-Asp
IKVAV	Ile-Lys-Val-Ala-Val
YIGSR	Tyr-Ile-Gly-Ser-Arg
Trk	tropomyosin receptor kinase
p75NTR	p75 neurotrophin receptor
PI3K	phosphoinositide 3-kinase
Akt	protein kinase B
ERK	extracellular signal-regulated kinase
PPy	polypyrrole
MWCNT	multi-walled carbon nanotube
IL-1Ra	interleukin-1 receptor antagonist
SPM	specialized pro-resolving mediator
FOXP3	forkhead box P3
PI	polyimide
PDMS	polydimethylsiloxane
ISO	International Organization for Standardization
RNA	ribonucleic acid
scRNA-seq	single-cell RNA sequencing
CODEX	co-detection by indexing
GFP	green fluorescent protein
*Snap25*	synaptosome-associated protein 25
